# Degradation of the α-Carboxyl Terminus 11 Peptide: *In Vivo* and *Ex Vivo* Impacts of Time, Temperature, Inhibitors, and Gender in Rat

**DOI:** 10.1021/acsptsci.4c00120

**Published:** 2024-04-22

**Authors:** Yagmur Tasdemiroglu, McAlister Council-Troche, Miao Chen, Benjamin Ledford, Russell A. Norris, Steven Poelzing, Robert G. Gourdie, Jia-Qiang He

**Affiliations:** † Department of Biomedical Sciences and Pathobiology, College of Veterinary Medicine, Virginia Tech, 225 Duck Pond Drive, Blacksburg, Virginia 24061, United States; ‡ Department of Medicine, Medical University of South Carolina, Charleston, South Carolina 29425, United States; § Center for Vascular and Heart Research, Fralin Biomedical Research Institute, Virginia Tech, 2 Riverside Circle, Roanoke, Virginia 24016, United States

**Keywords:** therapeutic peptide, αCT11, degradation, enzymatic inhibitors, rat, mass spectrometry

## Abstract

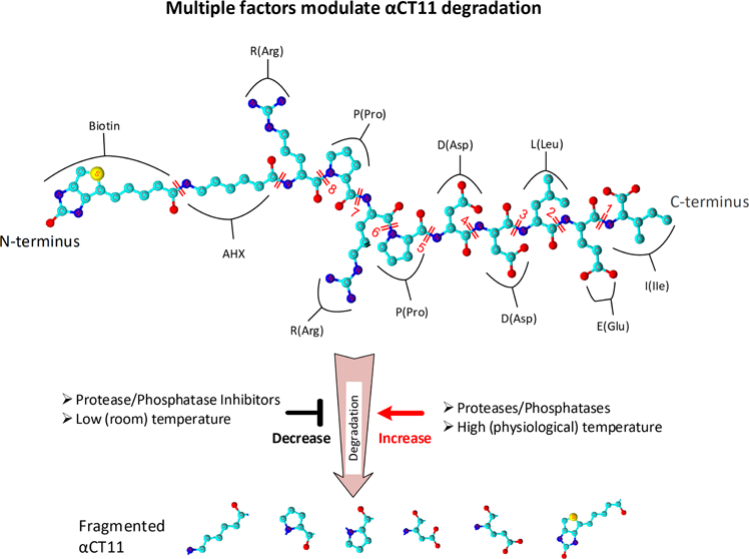

In previous research, a synthetic α-carboxyl terminus 1 (αCT1) peptide derived from connexin 43 (Cx43) and its variant (αCT11) showed beneficial effects in an *ex vivo* ischemia–reperfusion (I/R) heart injury model in mouse. In an *in vivo* mouse model of cryo-induced ventricular injury, αCT1 released from adhesive cardiac patches reduced Cx43 remodeling and arrhythmias, as well as maintained cardiac conduction. Whether intravenous injection of αCT1 or αCT11 produces similar outcomes has not been investigated. Given the possibility of peptide degradation in plasma, this study utilized *in vivo* I/R cardiac injury and *ex vivo* blood plasma models to examine factors that may limit the therapeutic potential of peptide therapeutics *in vivo*. Following tail vein administration of αCT11 (100 μM) in blood, no effect on I/R infarct size was observed in adult rat hearts on day 1 (D1) and day 28 (D28) after injury (*p* > 0.05). There was also no difference in the echocardiographic ejection fraction (EF%) between the control and the αCT11 groups (*p* > 0.05). Surprisingly, αCT11 in blood plasma collected from these rats was undetectable within ∼10 min after tail vein injection. To investigate factors that may modulate αCT11 degradation in blood, αCT11 was directly added to blood plasma isolated from normal rats without I/R and peptide levels were measured under different experimental conditions. Consistent with *in vivo* observations, significant αCT11 degradation occurred in plasma within 10 min at 22 and 37 °C and was nearly undetectable by 30 min. These responses were reduced by the addition of protease/phosphatase (PTase/PPTase) inhibitors to the isolated plasma. Interestingly, no significant differences in αCT11 degradation in plasma were noted between male and female rats. We conclude that fast degradation of αCT11 is likely the reason that no beneficial effects were observed in the *in vivo* I/R model and inhibition or shielding from PTase/PPTase activity may be a strategy that will assist with the viability of peptide therapeutics.

Globally, deaths from cardiovascular disease (CVD) increased from 12.4 million in 1990 to 19.8 million in 2022.^1^ It is predicted that the economic burden of CVD due to the loss of productivity between 2011 and 2025 will be $3.76 trillion for low- and middle-income countries.^2^ Notably, among all CVDs, ischemic heart disease remains the leading cause of CVD mortality.^1^ The most catastrophic manifestation of ischemic heart disease is acute myocardial infarction (MI), where the heart loses contractile function due to the lack of oxygen/nutrients and followed by cardiomyocyte death after an ischemic injury.^3,4^ Even though the early mortality rate from acute MI has been reduced through medical advancements, 12% of patients die within 6 months of an infarction, while 25% of patients develop heart failure (HF), leading to a 5-year mortality rate greater than 50% for these individuals.^5^

Presently, there are no curative approaches to treat patients with MI and/or HF.^6^ In the clinical management of acute MI, immediate treatments include the administration of antithrombotic drugs (e.g., heparin), blood thinners (e.g., aspirin), or the reopening of blocked coronary arteries (e.g., via angioplasty and stent placement).^7,8^ During the late stage of acute MI and HF, the primary treatment is to relieve patient symptoms, increase survival rate, and improve quality of life via administration of various drugs [e.g., classical angiotensin-converting enzyme (ACE) inhibitors, β blockers, diuretics,^9^ and digoxin^10^]. Even though these drugs are available to most patients, they are not a curative solution and do not completely restore heart function. In advanced stages of HF, implantation of medical devices (e.g., ventricular assist device or total artificial heart) or heart transplantation is often required.^9,11^

Development of effective new treatments for patients with MI and HF is an urgent unmet clinical need. Over the past decade, therapeutic peptides,^12,13^ stem cells [e.g., embryonic stem cells,^14^ induced pluripotential stem cells, fetal stem cells, adult stem cells^15^], stem-cell-derived somatic cells [e.g., cardiomyocytes,^15^ smooth muscle cells,^16,17^ endothelial cells^15^], stem cell transplantation in combination with biomaterials,^16,18−20^ miRNAs,^21^ gene therapy,^22^ xenotransplantation of hearts,^23^ and extracellular vesicles (EVs)^24^ have been tested in preclinical studies and clinical trials. However, these treatments have yet to advance the standard of care for acute MI and subsequent HF due to multiple reasons, including limited options for treatment administration, the instability of novel therapeutics [such as peptides^25^ and miRNAs *in vivo*(21)], and uncertainties about the clinical efficacy of cellular therapies, such as stem cell engraftment.^17^

Compared to stem cell and gene therapy, peptide-based treatments have shown promise for treating diseases, such as cardiovascular disorders, probably owing to their high affinity and specificity for their targets, as well as their relative safety and low number of side effects.^25^ For example, glucagon-like peptide-1 receptor agonists (GLP-1RA), such as *Exenatide* and *Liraglutide*, are both synthetic peptides that mimic the function of gastrointestinal peptides that stimulate glucose-dependent insulin release from pancreatic β-cells.^26^ These peptidic drugs have shown beneficial effects in patients with diabetic HF with the underlying mechanism likely being via controlling blood glucose levels and promoting weight loss, thereby indirectly improving cardiac function.^27,28^

Connexin 43 (Cx43), α-carboxyl terminal (CT) mimetic peptide 1 [αCT1], is a 25-amino acid (AA) peptide containing an antennapedia sequence attached to the CT-most 9 amino acids of Cx43.^29^ Following three phase II clinical trials in humans, αCT1 is presently in phase III testing for cutaneous radiation injury.^13,30,31^ Interestingly, both αCT1 and its shorter derivative αCT11 (a 9-AA variant of αCT1 without the antennapedia sequence) have shown utility in preclinical mouse studies for cardioprotection from ischemic injury.^29^ In this study, it was determined that preischemic perfusion of αCT1 or pre- and -postischemic perfusion of αCT11 significantly preserved cardiac contractile function in isolated mouse hearts perfused *ex vivo* following a 20 min ischemia/40 min reperfusion (I/R) injury.^29^

These findings suggest that peptides based on the Cx43 CT have the potential to be used to treat patients with MI and HF. However, whether intravenous administration (e.g., via tail vein of rat or mouse) of αCT11 generates the same or similar beneficial effects on MI or HF has not been tested in intact animal models.

Thus, the present study aims to (1) examine whether *in vivo* intravenous administration of αCT11 can prevent or reduce infarct size and increase cardiac ejection fraction (EF%) in a rat model of acute MI and (2) probe the “pharmacokinetic” properties of the peptide following intravenous injection in a rat *in vivo* I/R injury model and after addition to isolated blood plasma. The overarching objectives of the study are to provide insight into factors that may affect αCT11 degradation in body fluids, such as blood, as well as identify strategies that increase the stability of αCT11 (and other peptides) *in vivo*. The ultimate goal of this study is to translate this therapeutic peptide to clinical trials for the treatment of MI and HF.

## Materials and Methods

### Animals

Adult Sprague–Dawley rats, both male (22) and female (22), aged 12–15 weeks, from Charles River (strain code: 001) were used in this study. Rats were individually labeled using ear tattoos^32^ and housed in the Animal Facility of the Virginia-Maryland Regional College of Veterinary Medicine at Virginia Tech. The animals had free access to standard rodent chow and water. Ambient temperature and relative humidity were kept at 22–23 °C and 50–70%, respectively. Room illumination was automatically set on a 12 h day/night light cycle, with lights on at 7:00 am and off at 7:00 pm. All animal procedures were approved by the Institutional Animal Care and Use Committee of Virginia Tech and conformed to the National Institutes of Health Guide for the Care and Use of Laboratory Animals.

### Reagents

The major reagents and materials in this section are grouped and listed together based on vendor names. Suture 8-0, Cat. XXS-N807T6, and suture 5-0, Cat. M-G518R19-U, were purchased from AD Surgical (Sunnyvale, CA). αCT11 was synthesized by the American Peptide Company (Sunnyvale, CA). Protease inhibitor cocktail [including AEBSF, aprotinin, bestatin, E64, leupeptin, and pepstatin A, also see Table S4], Cat. B14001, and phosphatase inhibitor cocktail (including *p*-bromotetramisole oxalate, cantharidin, imidazole, microcystin LR, sodium molybdate, sodium orthovanadate, sodium tartrate, and sodium fluoride; also see Table S4), Cat. B15001, were from Bimake (Houston, TX). Ethiqa XR (Cat. 099114), heparin (Cat. 038213), isoflurane (Cat. 502017), ketoprofen (Cat. 002800), and saline (Cat. 510224) were from MWI Veterinary Supply (Boise, ID). Formalin (10%) (Cat. HT501128-4L), potassium chloride (KCl) (Cat. P9541-500G), and triphenyltetrazolium chloride (TTC) (Cat. T8877-50G) were from Sigma-Aldrich (St. Louis, MO). Acetonitrile (ACN) (Cat. A955-4), formic acid (FA) (Cat. AC270480010), and Parker Aquasonic Clear Ultrasound Gel (Cat. 5067723) were from ThermoFisher Scientific (Waltham, MA). In addition, 0.5 mL (Cat. 1405-2600) and 2.0 mL (Cat. CC7682-7596) low-adhesion microcentrifuge tubes were from USA Scientific (Ocala, FL). 1.8 mL amber vials (Cat. 46610-0726), insert (Cat. 97051-408), and cap (Cat. 89239-022) were all from VWR (Radnor, PA). Watters HSS T3 column (Cat. 186003539) and matching VanGuid column (Cat. 176004352) were from Waters Corp (Milford, MA).

### Rat Model of I/R MI and Tail Vein Injection of αCT11

After body weight assessment, rats (male only) were anesthetized in an induction chamber using 3–5% isoflurane supplied by a V3000PK isoflurane vaporizer (Parkland Scientific, Coral Springs, FL), and the animals were then mounted on a custom-made intubation stand and maintained with 1–2% isoflurane through a custom-made non-rebreathing circuit covering the animal’s nose. Under the guidance of a small animal incubation device (Braintree Scientific, Braintree, MA), an endotracheal tube (e.g., 16G intravenous plastic catheter) was carefully inserted into the trachea via the ostium of the trachea, which can be seen from the animal’s mouth. Rats were then transferred to and fixed on a surgical bed heated at 37 °C using a TC-1000 temperature controller (Palmer, PA), where body temperature was automatically maintained by the temperature controller through a vaseline-lubricated probe placed in the animal’s rectum. Once the catheter was connected to a TOPO small animal ventilator (Kent Scientific, Torrington, CT) coupled to the isoflurane vaporizer, a synchronous movement of the animal’s chest with the click sound of the ventilator indicated successful intubation.

The acute MI model of I/R was induced as in previously published methods.^33,34^ Briefly, after the hair on the animal’s chest was removed using nair remover lotion (Church & Dwight, Ewing Township, NJ) and administration (s.q.) of 0.65 mg/kg Ethiqa XR (an analgesic drug) and 10 mg/kg ketoprofen (a nonsteroidal anti-inflammatory drug “NSAID”), the chest was surgically opened between the third and fourth intercostal space to expose the heart. Thereafter, a 8-0 suture was passed underneath the left anterior descending (LAD) coronary artery and tied to a tiny plastic tube [∼3–4 mm length (*L*) × ∼1–2 mm outside diameter (OD)] to temporarily block blood flow. After 40 min of blockage, the suture was removed to allow blood reperfusion and the chest was closed using a 5-0 suture.

Immediately after, 10 mM αCT11 was injected via the tail vein to generate an estimated final concentration of 100 μM in the plasma (the injected volume was calculated based on the percentage of blood volume against the body weight of each rat). Control rats were injected with the same amount of saline only. Fully recovered animals were then transferred to the animal facility and housed, as described above. Ethiqa XR and ketoprofen were administrated accordingly up to 6 days postsurgery.

### Echocardiography Measurement

A series of echocardiographs were recorded immediately before and after [on day 1 (D1), D14, and D28] MI surgery to evaluate cardiac function (i.e., EF%) using ultrasound gel and a Vevo 2100 system (Fujifilm VisualSonics, Toronto, Canada) equipped with an MS400 transducer (18–38 MHz). The EF% and other parameters (data not shown) were calculated using the software installed on the system. At the end of the experiments, animals were euthanized on D1 or D28 after infarction by tail vein injection of 3 molarity (M) KCl to stop the heart in the diastolic phase.^33^ Hearts were then extracted for postpathohistological analyses and were carried out as described below.

### TTC Staining of Infarcted Cardiac Slices

The extracted hearts were frozen at −20 °C for 5–10 min and transversally cut in a rat heart slicer matrix (Zivic Instruments, Pittsburgh, PA) at 2 mm thickness starting from the apex and followed by incubation in the water-soluble TTC solution in the dark for 20–30 min at 37 °C. Upon completion of TTC staining, viable tissues were stained red, while nonviable tissues were stained yellow(ish).^35^ Slices were then fixed with 10% phosphate-buffered formalin at room temperature (RT) for 30 min and directly photographed using an Olympus IX73 DP70 camera (Olympus Americas, Breinigsville, PA).^35^ Infarct size was measured and calculated using ImageJ v1.53 (NIH, Bethesda, MD) according to the published method.^33^

### *In Vivo* “Pharmacokinetic” Analysis of αCT11

A second group of rats (only male) was not subjected to MI but injected (via the tail vein) with the same concentration of αCT11 as described in the Rat Model of I/R MI and Tail Vein Injection of αCT11 section. After injection, about 250–350 μL of blood was withdrawn at 1, 5, and 10 min following administration and transferred into an anticlotting tube containing heparin. Plasma was then separated at 4 °C and 2000*g* for 10 min using an Eppendorf Centrifuge 5810R (Eppendorf North America, Framingham, MA) and stored at −80 °C or used immediately for liquid chromatography tandem mass spectrometry (LC/MS-MS; described in the Prepreparation and Measurements of Plasma Containing αCT11 for LC/MS-MS Analysis section).

### *Ex Vivo* Degradation Analysis of αCT11 in Rat Plasma

A third group of rats (including both males and females) was not subjected to either MI or the tail vein injection of αCT11. Instead, after injection of heparin (300 U/mL blood, the total units of heparin were estimated from body weight of each rat), as much blood as possible was collected under deep anesthesia via a cardiac puncture with a 21G needle connected to a syringe containing heparin. Blood was then transferred into an anticlotting tube on ice, followed by plasma separation as described above. All plasma collected from either males or females were randomly sorted into 4 groups (G) per gender [G1: 22 °C; G2: 37 °C, G3: 22 °C + inhibitor cocktail and G4: 37 °C + inhibitor cocktail] and incubated at 22 °C [in an Eppendorf thermomixer (Eppendorf North America, Framingham, MA)] or at 37 °C [in a digital dry bath (Benchmark, Sayreville, NJ)] with or without additions of αCT11 (at a final concentration of 100 μM in each tube) in the presence or absence of a PTase/PPTase inhibitor cocktail (at a final concentration of 1.5× of the original concentration). It took less than ∼30 s for the 22 °C groups to reach 22 °C and ∼75 s for the 37 °C groups to reach 37 °C. During intubation, 100 μL of plasma was taken in triplicate from the tube of each animal at 0, 5, 30, and 60 min following additions of αCT11 and the cocktail inhibitors (if applied). The collected samples were then transferred to new tubes for further preparation prior to LC/MS-MS analysis.

### Prepreparation and Measurements of Plasma Containing αCT11 for LC/MS-MS Analysis

Plasma samples collected from either *in vivo* (see the In Vivo “Pharmacokinetic” Analysis of αCT11 section) or *ex vivo* (see the Ex Vivo Degradation Analysis of αCT11 in Rat Plasma section) groups were processed in the same way outlined here, according to the modified protocol from a previous publication.^36^ Briefly, 100 μL of plasma was added to a 0.5 mL low-adhesion microcentrifuge tube containing 4% FA in 200 μL of ACN [see Table S1]. The tubes were slightly (∼1 min) vortexed and centrifuged for 5 min at 11,332*g* using an Eppendorf Centrifuge 5424R (Eppendorf North America, Framingham, MA) to recover the supernatants. 100 μL of the supernatant was then combined with 100 μL of deionized H_2_O in 2 mL amber autovials with a low-volume insert and capped. The samples were either stored at 4 °C up to 4 weeks (see the Results section) or analyzed immediately using a Waters H-Class UPLC mass spectrometer (Milford, MA) and its accompanying MassLynx software, version 4.2 (Milford, MA).

For measurement, the sample extracts were first separated chromatographically with an HSS T3 column and matching VanGurd column at 40 °C. A 4 μL sample was injected onto the column using a refrigerated autosampler at 5 °C. Mobile phase A was comprised of 1% FA in H_2_O and mobile phase B was comprised of 1% FA in ACN (Table S1). The mobile phase was delivered to the column at a flow rate of 0.4 mL/min. The gradient elution program is listed in Table S1. Tuning was performed on each analyte by direct infusion of a standard solution (0.1 ng/μL) at a rate of 10 μL/min. The parent and production transitions for αCT11 peptide are shown in Table S2, while the mass spectrometry parameters used for detecting αCT11 are listed in Table S3.

### Data Analysis and Statistical Testing

Data were collected at 0 min in triplicate from each group using LC/MS-MS and were first calibrated as 100%; all other data from the same group were then normalized as % change over the data at 0 min. A Student’s *t*-test was used for comparison of 2 groups, while 1- or 2-way ANOVA followed by Tukey’s test was used for comparisons of ≥3 groups with 1 or 2 factors. All data were expressed as mean ± standard error (SE), if not otherwise specified, with *p* < 0.05 considered statistically significant. Data were analyzed and plotted using GraphPad Prism 9.3.1 (San Diego, CA) and Microsoft 2019 MS Excel (Redmond, WA).

## Results

### Establishment of a Mass Spectrum-Based Method for Detecting αCT11 in Rat Plasma

**Figure 1:**
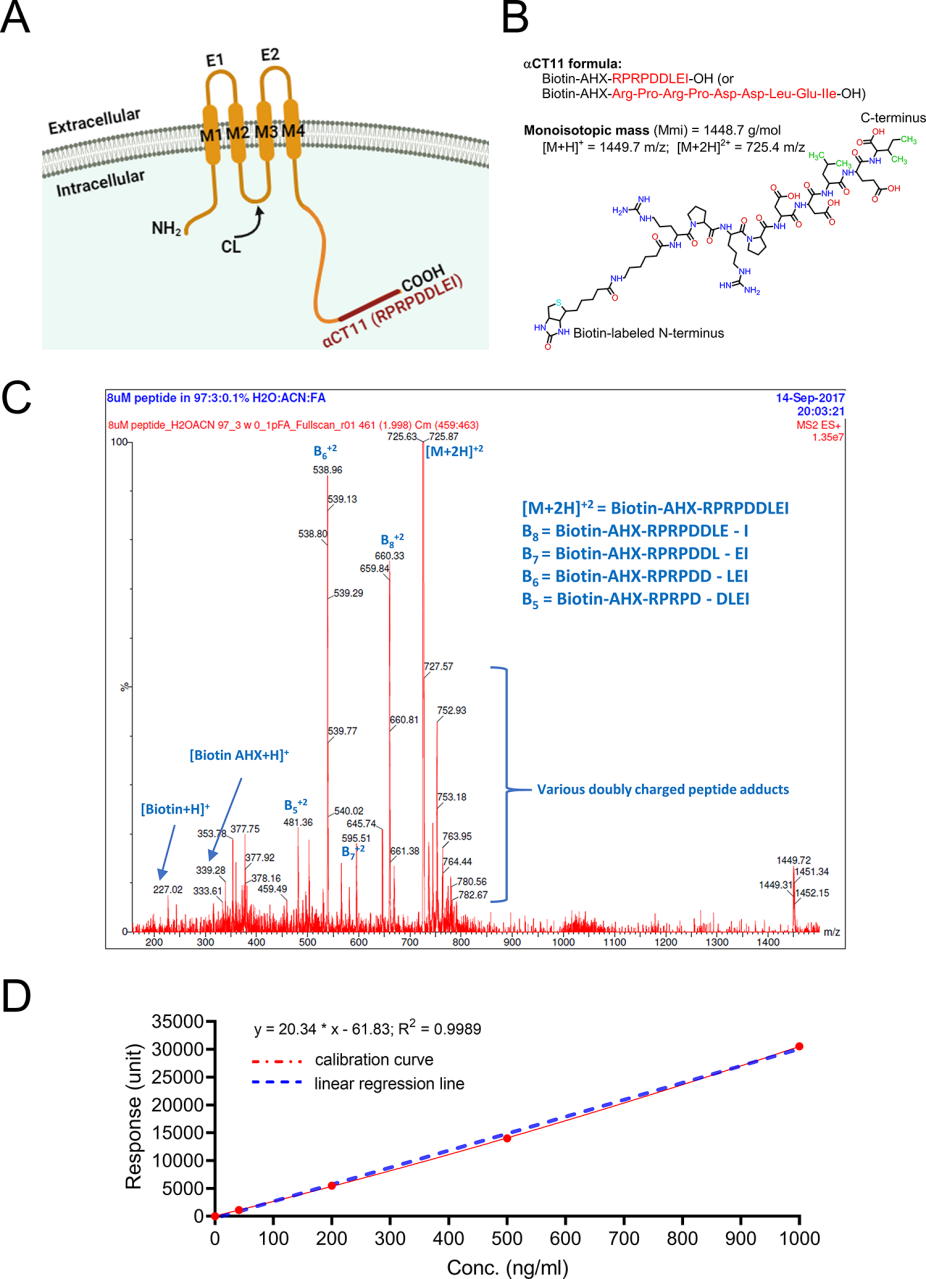
Basic structure of Cx43 and detection of αCT11 with LC-MS/MS. (A) A diagram of Cx43, comprised of M1 to M4, E1 to E2, one CL, N-terminus, and C-terminus bound with αCT11 (9-AA, red line). (B) αCT11 molecular structure tagged with biotin at the N-terminus. (C) Full scan mass spectrum of the αCT11 peptide from 150 to 1500 *m*/*z*. *Y*-axis is scaled to the peak of 539 *m*/*z* to better highlight the peak of 1449.7 *m*/*z*. Peptide fragments are labeled according to matches using ProteinProspector developed by the University of California San Francisco (https://prospector.ucsf.edu/prospector/mshome.htm). (D) The calibration curve was generated with 5 concentrations (red dots) and used to normalize the plasma concentration of αCT11 with LC-MS/MS. A linear regression fit is shown by the blue dashed line. Panels (A) and (B) were originally created by the authors using the online software BioRender (https://biorender.com) and ACD/ChemSketch V2021.2.0 (https://www.acdlabs.com), respectively. Abbreviations: αCT11: α-carboxyl terminus 11; Conc.: concentration; C-terminus: carboxylic-terminus; CL: cytoplasmic loop; Cx43: connexin 43; AHX: 6-aminohexanoic acid; D (Asp): aspartic acid; E (Glu): glutamic acid; E1 to E2: extracellular domains 1 and 2; I (IIe): Isoleucine; L (Leu): leucine; LC-MS/MS: liquid chromatography with tandem mass spectrometry; M1 to M4: transmembrane domain 1 to 4; N-terminus: nitrogen-terminus; P (Pro): proline; R (Arg): arginine.

αCT11 is a 9-AA peptide tagged with a biotin molecule at its N-terminus and has a molecular weight (MW) of 3597.3 Da (D) (Figure 1A,B). Using LC-MS/MS, we detected all fragments of αCT11 within the mass spectra from 150 to 1500 *m*/*z* (Figure 1C). The concentrations of αCT11 were determined using a standard curve with a coefficient of determination (*R*^2^) of >0.99 (Figure 1D). The limit of detection (LOD) of the system was approximately 3 ng of αCT11/mL of plasma, given that the signal-to-noise ratio was 3. The limit of quantification (LOQ) was considered the lowest concentration on the calibration curve and was ∼12 ng of αCT11 per mL of plasma.

### αCT11 Did Not Reduce Infarct Size Likely Due to Its Rapid Degradation in Blood Following Tail Vein Injection

**Figure 2:**
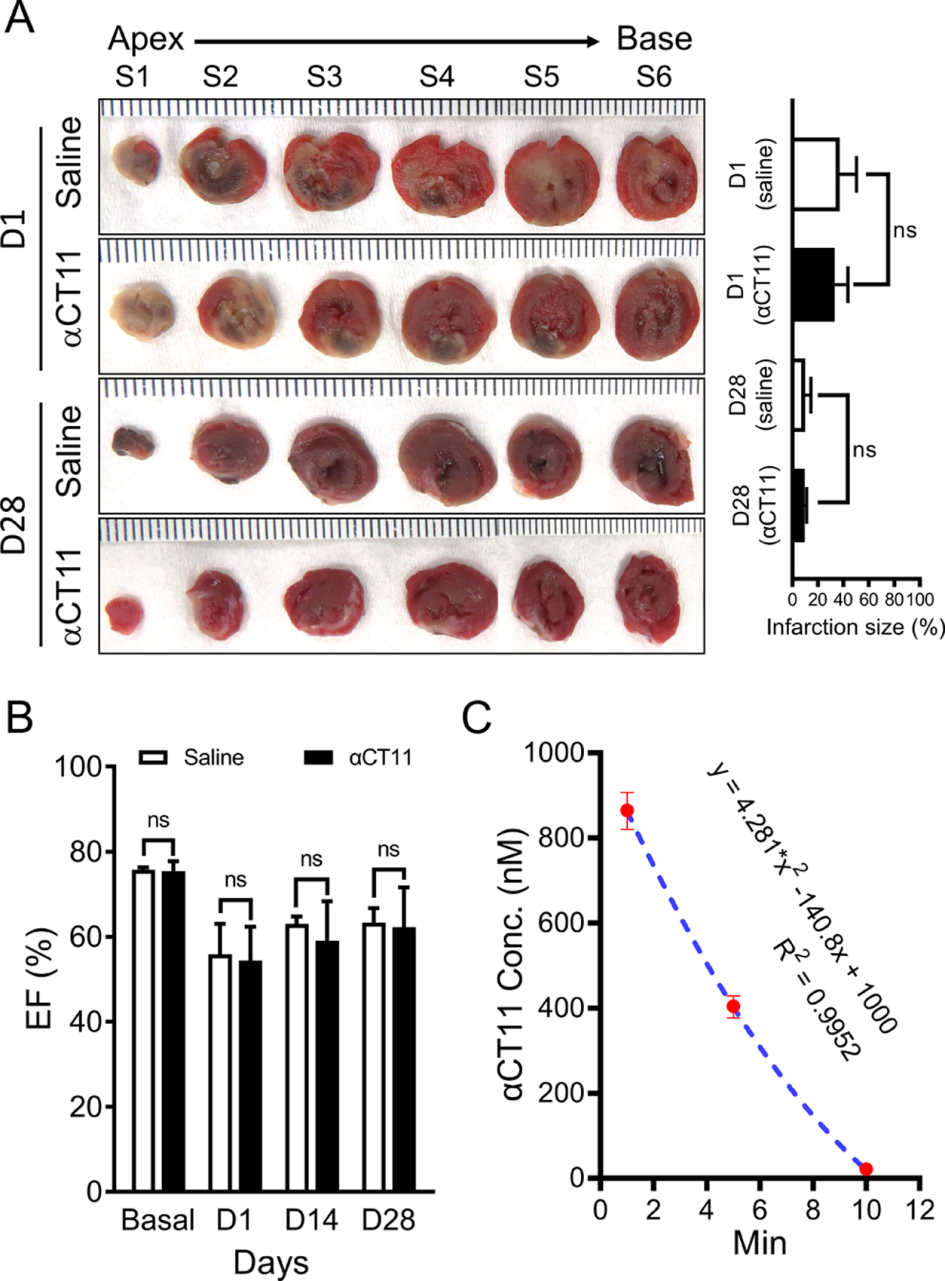
Intravenous injection of αCT11 failed to rescue cardiac function following acute MI in rat due to rapid degradation *in vivo.* (A) TTC staining of rat cardiac sections on D1 and D28 after myocardial infarction induced by 40 min ligation of the LAD coronary artery and immediate injection of αCT11 peptide via the tail vein. Healthy cardiac tissues are shown in red, while the infarcted tissues are shown in yellow/white or dark brown (due to hemorrhage) after TTC staining. On each section (2 mm thickness), the area sizes of both infarcted and total area were measured using NIH ImageJ and the % changes of infarction size vs total area were plotted on the right side (*n* = 3–4 male rats; ns). (B) The mean values of EF% collected before and on D1, D14, and D28 after infarction are shown as bar graphs. (C) To determine the *in vivo* degradation of αCT11, peptide dissolved in saline was injected into different groups of rats via the tail vein, and blood was then withdrawn at 2, 5, and 10 min (the data in 30 and 60 min are not shown) after injection. Plasma concentrations of αCT11 were measured using LC-MS/MS (*n* = 3–4 male rats). A polynomial (quadratic) fit is shown as a blue dashed line (*R*^2^ = 0.9952). Two-way ANOVA was used for statistical testing in both panels (A) and (B). Abbreviations: D1, D14, and D28: days 1, 14, and 28 after infarction; EF(%): ejection fraction (%); LAD: left anterior descending; ns: no significance. S1 to S6: Section 1 (apex) to 6 (base); TTC: 2,3,5-triphenyltetrazolium chloride. See Figure 1 for other abbreviations defined previously.

Our previous study indicated that an infusion of αCT11 (10–50 μM of the final concentration in the perfusion buffer for 20 min) exerted significant cardioprotection in an *ex vivo* I/R model of mouse heart.^29^ To further study this effect, an *in vivo* rat model of MI was used, and αCT11 was injected (via tail vein) immediately after chest closing. The injected amount of αCT11 was calculated based on the animal’s body weight (and blood volume) to reach a final concentration of 100 μM in the blood circulation. No statistical difference in infarct sizes was found between αCT11-treated and control animals (Figure 2A, ns, *n* = 3–4). The mean infarct sizes were 37.1 ± 13% in the control vs 33.3 ± 10% in the αCT11-treated group on D1 (nanoseconds, *n* = 3) and 9.6 ± 2.9% in the saline vs 9.7 ± 1.6% in the treated group on D28 (nanoseconds, *n* = 4–5) (Figure 2A). Echocardiograhically assessed EFs (%) were also comparable between the control and the peptide-treated groups at the two postinfarct time points (Figure 2, ns, *n* = 3–6), suggesting that the peptide did not protect the injured heart under the experimental setting tested.

To investigate the potential reasons underlying the inconsistent results between the *ex vivo* (Langendorff perfused heart^29^) and *in vivo* experiments, αCT11, with the same final concentration of 100 μM, was directly injected into rats without MI via the tail vein. Blood was then periodically collected to measure αCT11 levels at different time points. Figure 2C indicates that αCT11 quickly degraded in the blood circulation following the injection. At about 5 min postinjection, there was a ∼53% reduction of the initial αCT11 concentration, and the peptide was almost undetectable 10 min postinjection (*n* = 3), implying that factors (e.g., proteases) in the plasma efficiently promoted αCT11 degradation.^25^

### *Ex Vivo* Time-Dependent Degradation of αCT11 in Rat Plasma

**Figure 3:**
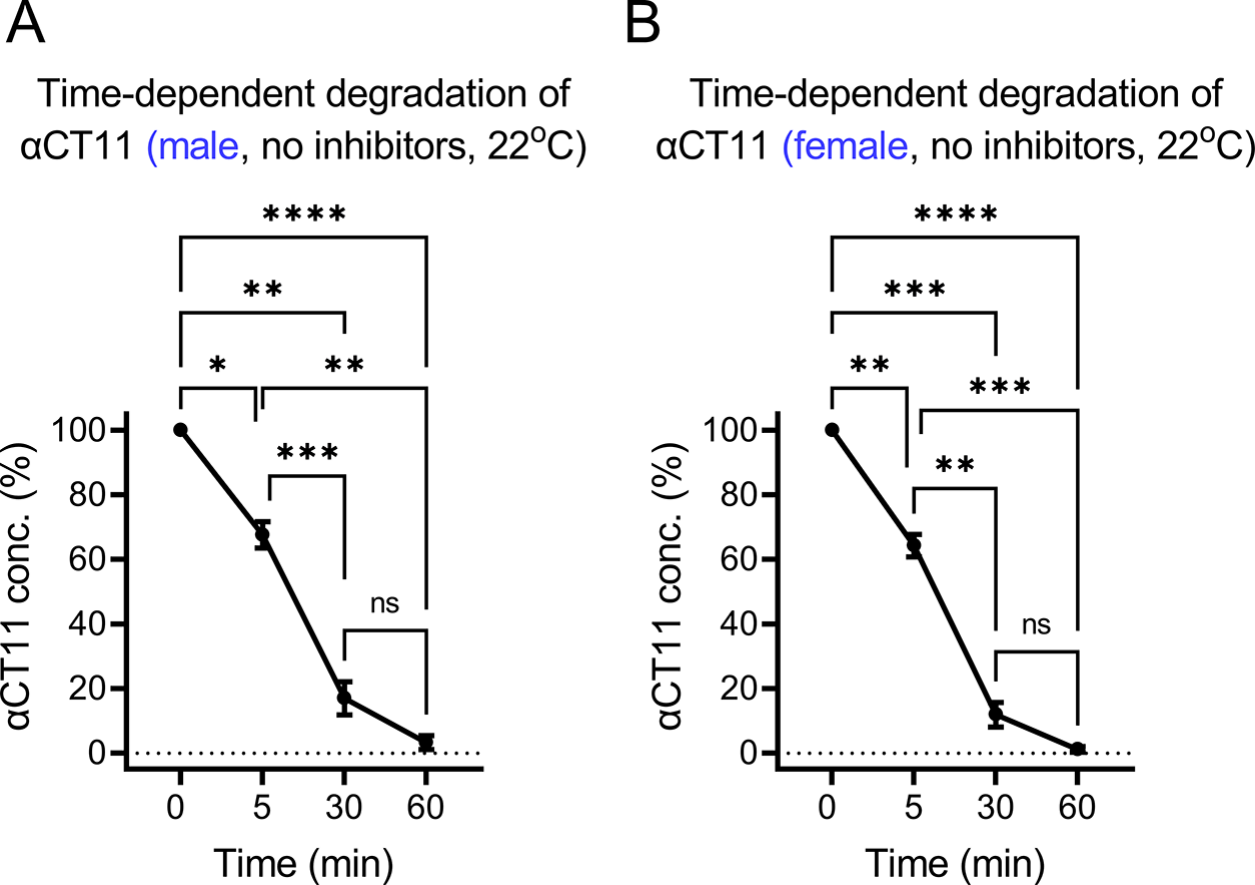
*Ex vivo* time-dependent degradation of αCT11 in isolated rat plasma. (A, B) Blood was directly collected from the left ventricular chambers of male (left) and female (right) rats under deep anesthesia. After being separated from blood cells using a 4 °C centrifuge, the resulting cold plasma was then transferred into different tubes, and αCT11 was added to reach a final concentration of 100 μM in the absence of the protease/phosphatase inhibitor cocktail. The samples were then maintained at 22 °C (on a tube heater) for 0, 5, 30, and 60 min, followed by measurements of the plasma αCT11 concentrations using LC-MS/MS. Results were plotted as the percent change (%) normalized to those at 0 min. One-way ANOVA was used for statistical testing. In both panels (A) and (B), a total of 9 replicates from 3 rats in each group were analyzed. ns: no significant; *p < 0.05; **p < 0.01; ***p < 0.001, ****p < 0.0001. See Figures 1 and 2 for abbreviations defined previously.

To dissect factors that affect αCT11 stability in the blood, we examined the time-dependent decomposition of the peptide using an *ex vivo* model of normal rat plasma. The results showed that the percentage of αCT11 degradation rapidly increased at 22 °C (i.e., room temperature, RT), decreasing from 100% in the starting concentration to 67.6 ± 4.1% (*p* < 0.05), 17.0 ± 5.1% (*p* < 0.001), and 3.2 ± 2.2% (*p* < 0.0001) at 5, 30, and 60 min, respectively, in male plasma (Figure 3, left panel, *n* = 3); in female plasma, similar trends were found with concentrations starting at 100% of the beginning concentration and decreasing to 64.3 ± 2.0% (*p* < 0.01), 11.9 ± 2.2% (*p* < 0.001), and 1.3 ± 0.5% (*p* < 0.0001) at 5, 30, and 60 min, respectively (Figure 3, right panel, *n* = 3). These data indicate that significant time-dependent degradation of αCT11 takes place, even at RT.

### High (Physiological) Temperature Speeds Up αCT11 Degradation

**Figure 4:**
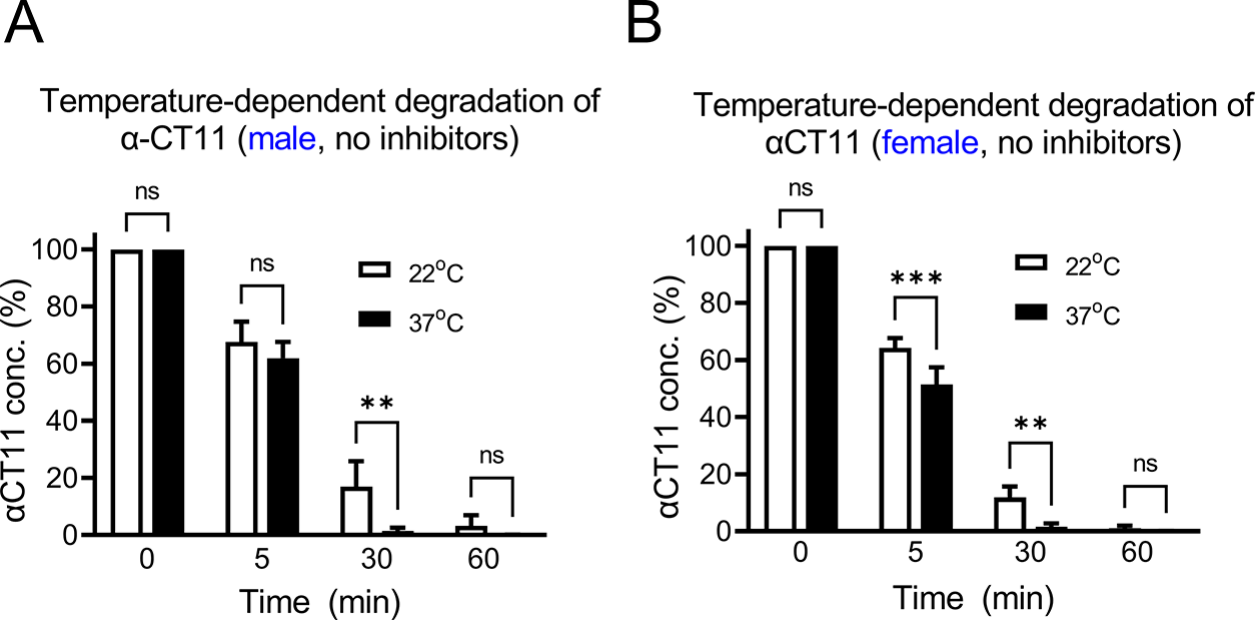
Temperature-dependent degradation of αCT11 in isolated rat plasma. (A, B) Blood was collected from the left ventricular chambers of male (left) and female (right) rats under deep anesthesia. After being separated from blood cells using a 4 °C centrifuge, the resulting plasma was transferred into different tubes, and αCT11 peptide was added to reach a final concentration of 100 μM in the absence of protease/phosphatase (PTase/PPTase) inhibitor cocktails. Samples were then maintained at 22 or 37 °C (on a tube heater) for 0, 5, 30, and 60 min, followed by measurements of the plasma αCT11 concentration using LC-MS/MS. The results were plotted as a percent change (%) and normalized to those at 0 min for each temperature. In both panels (A) and (B), a total of 9 replicates from 3 rats in each group were analyzed. Two-way ANOVA was used for statistical testing. ns: no significant; **p < 0.01; ***p < 0.001. See Figures 1–3 for abbreviations defined previously.

To examine the effects of physiological temperature on αCT11 degradation, isolated plasma samples with added αCT11 were analyzed after 0, 5, 30, and 60 min incubation at 37 °C. The results showed that 37 °C significantly promoted αCT11 degradation relative to that observed at RT. In male plasma, the levels of αCT11 dropped from 67.5 ± 4.1% (22 °C) to 61.89 ± 5.7% (37 °C) at 5 min (ns), from 16.9 ± 8.9% (22 °C) to 1.5 ± 1.2% (37 °C) at 30 min (*p* < 0.01), and from 3.2 ± 3.7% (22 °C) to 0.04 ± 0.06% (37 °C) at 60 min (ns) (Figure 4 left panel, *n* = 3). Similar degradation patterns were observed in female plasma, where the levels of αCT11 dropped from 64.3 ± 2.0% (22 °C) to 51.4 ± 3.5% (37 °C) at 5 min (*p* < 0.001), from 11.9 ± 2.2% (22 °C) to 1.6 ± 0.6% (37 °C) at 30 min (*p* < 0.01), and from 1.1 ± 0.5% (22 °C) to 0.1 ± 0.03% (37 °C) at 60 min (ns) (Figure 4 right panel, *n* = 3). These data imply that αCT11 stability in blood plasma is greatly reduced at a physiological body temperature.

### Inhibition of PTases/PPTases Significantly Reduced αCT11 Degradation

**Figure 5:**
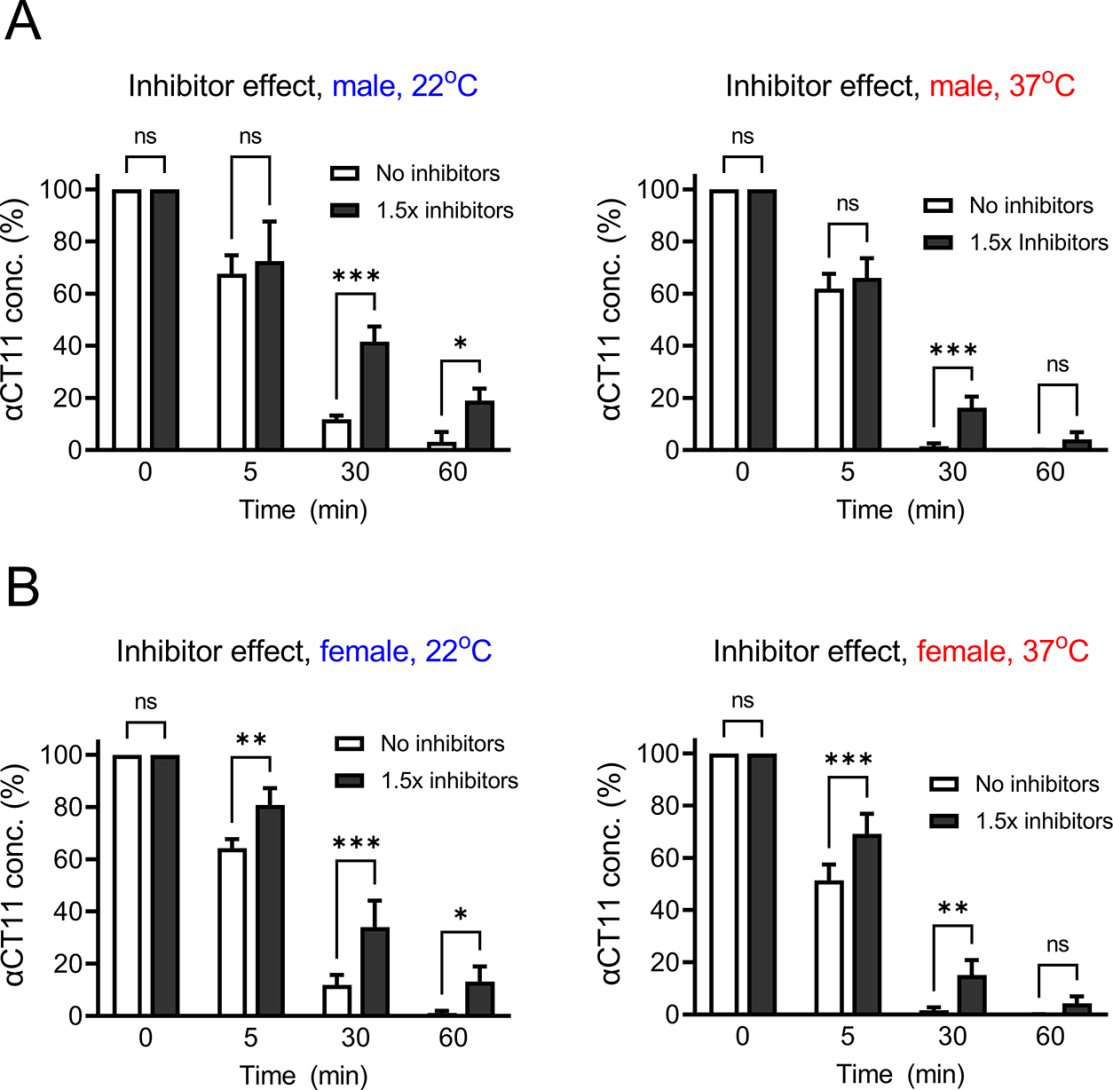
PTase/PPTase inhibitor cocktails slowed down the αCT11 degradation, even at physiological temperature. (A, B) Blood was collected from the left ventricular chambers of male (top panel) and female (bottom panel) rats under deep anesthesia. After being separated from blood cells using a 4 °C centrifuge, the resulting plasma was transferred into different tubes, and αCT11 was added to reach a final concentration of 100 μM in the presence or absence of 1.5× PTase/PPTase inhibitor cocktail. The samples were then maintained at 22 °C (left panel) or 37 °C (right panel) for 0, 5, 30, and 60 min, followed by measurements of plasma αCT11 using LC-MS/MS. The results were plotted as percent change (%) normalized to those at 0 min under each temperature. In both panels (A) and (B), a total of 9 replicates from 3 rats in each group were analyzed. Two-way ANOVA was used for statistical testing. ns: no significant; *p < 0.05; **p < 0.01; ***p < 0.001. See Figures 1–4 for abbreviations defined previously.

PTases/PPTases are known to proteolyze many proteins and peptides.^25,37,38^ To examine the potential protective effects of proteases/phosphatase inhibitors (PPIs) on αCT11, we^39^ tested a cocktail of inhibitors that consisted of 6 specific protease antagonists and 8 specific antagonists of phosphatases (also see Table S4) at 1.5× concentration, as determined by a preliminary study of the effectiveness of inhibition. The inhibitor cocktail significantly retarded αCT11 degradation in male plasma at 22 °C, following 30 min incubation [the percentage of αCT11 was 11.9 ± 1.1% (no inhibitors) vs 41.6 ± 3.3% (with 1.5× inhibitors) (Figure 5A, *n* = 3, *p* < 0.01)] and 60 min incubation [the percentage of αCT11 was 3.2 ± 2.2% (no inhibitors) vs 19.1 ± 2.7% (with 1.5× inhibitors) (Figure 5, left panel, *n* = 3, *p* < 0.05)]. Similar patterns of improved αCT11 stability in the presence of PTase/PPTase inhibitors were also observed at 37 °C (Figure 5, right panel, *n* = 3). Although no statistical difference was found at 60 min, αCT11 levels appeared to be higher in the groups treated with 1.5× inhibitors than those without inhibitors.

Interestingly, the female group’s plasma appeared to show a modest increase in sensitivity (at the 5 min time point) to the cocktail of inhibitors relative to males. At 22 °C, 1.5× inhibitors significantly slowed down the degradation of αCT11 at all time points after 0 min, where the percentage of αCT11 was 64.2 ± 2.0% (no inhibitors) vs 80.9 ± 3.7% (with 1.5× inhibitors, *p* < 0.01) at 5 min, 11.9 ± 2.2% (no inhibitors) vs 34.0 ± 5.9% (with 1.5× inhibitors, *p* < 0.001) at 30 min, and 1.1 ± 0.5% (no inhibitors) vs 13.2 ± 3.3% (with 1.5× inhibitors, *p* < 0.05) at 60 min (Figure 5B left panel, *n* = 3). Even at 37 °C, the percentage of αCT11 was still higher in treated groups with 1.5× inhibitors than the control groups with 51.4 ± 3.5% (no inhibitors) vs 69.2 ± 4.4 (with 1.5× inhibitors, *p* < 0.001) at 5 min and 1.6 ± 0.6% (no inhibitors) vs 15.1 ± 3.3% (with 1.5× inhibitors, *p* < 0.01) at 30 min. Although no statistical difference was found at 60 min at 37 °C, the percentage of αCT11 appeared higher in the treated group than in the control group (Figure 5B, right panel, ns, *n* = 3). Together, these data suggest that cocktail inhibitors slowed down the αCT11 degradation in both males and females at either temperature, and the effects of inhibitors on αCT11 degradation appeared comparable between the two genders at 30 and 60 min.

### Males and Females Showed Similar Patterns in αCT11 Degradation

To examine whether gender modulates the degradation of αCT11, we compared the levels of αCT11 between male and female rats under the same experimental conditions as those described above (i.e., the same temperature, inhibitors, and time points). Figure S1 shows that in the absence and presence of inhibitors, there were no significant differences in αCT11 concentrations between the two genders at any time point under either temperature (Figure S1, ns, *n* = 3). Although αCT11 degradation in female rats was reduced only at 5 min with 1.5× inhibitors compared to its control at both temperatures (Figure 5B), direct comparisons of αCT11 levels did not reach significant differences between male and female rats at any given time point (Figure S1). Overall, these data suggest that gender might not have a significant effect on αCT11 degradation in the *ex vivo* model.

### Mass Spectrum Samples Containing αCT11 Can Be Stably Stored at 4 °C Up to 4 Weeks

To assess the stability of plasma samples containing αCT11 prepared for mass spectrum analyses, male rat blood samples were kept at 4 °C in a refrigerator for 1, 4, and 6 weeks immediately after being initially tested at their designated time points. As shown in Figure S2, no statistically significant differences in αCT11 concentration were observed between week 0 vs week 1 and week 0 vs week 4 under the same temperature (22 or 37 °C), with or without inhibitors. However, samples that were incubated at 37 °C showed significant decreases in αCT11 concentration after 6 weeks of storage even at 4 °C in the presence of 1.5× inhibitors (Figure S2C, *p* < 0.001, *n* = 3). These results indicate that the mass spectrum samples can be stored at 4 °C for up to 4 weeks without significant degradation of αCT11.

## Discussion

**Figure 6:**
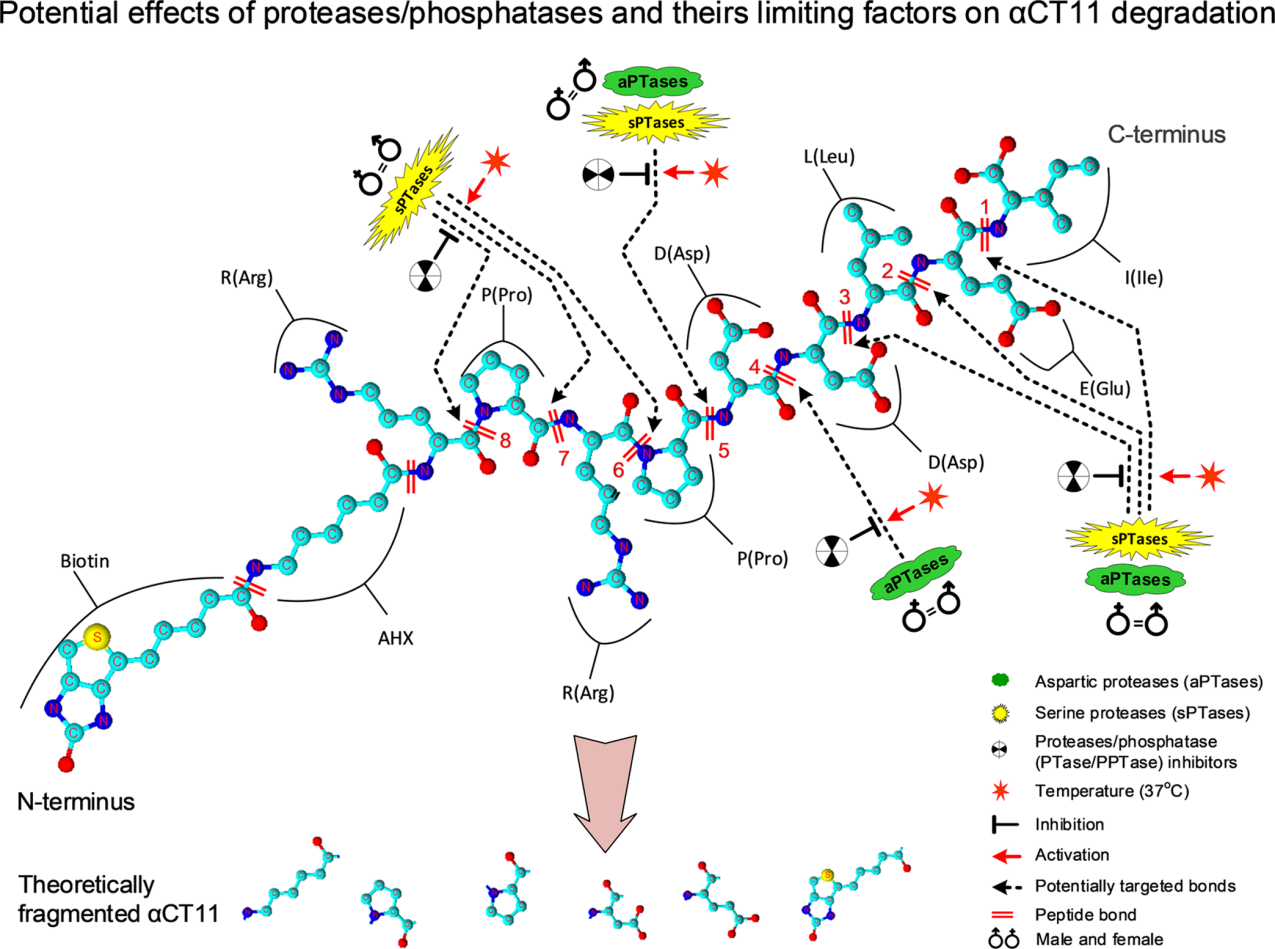
Working hypothesis of *ex vivo* and *in vivo* degradation of αCT11 in rat plasma. αCT11 peptide contains 8 bonds (labeled with short parallel lines in red) between the AAs. Starting from the C-terminus, bonds #1, #2, #3, and #5 are presumed to be targeted by both aspartic PTases (aPTases) and serine PTases (sPTases), whereas bond #4 is presumed to be targeted by an aPTase and the rest of the bonds (#6, #7, and #8) are presumed to be hydrolyzed by sPTases (also see Table S2). Meanwhile, the enzymatic activities of PTases and PPTases can be regulated by PTase/PPTase cocktail inhibitors and the temperature. Note: the colored molecular structure of αCT11 was created using ACD/ChemSketch (https://www.acdlabs.com). See Figures 1–5 for other abbreviations defined previously.

Utilizing both an *in vivo* rat MI model and an *ex vivo* model of isolated blood plasma, we investigated potential reasons for the failure of αCT11 to provide cardioprotection from I/R injury following tail vein administration of the peptide (Figure 2). In the *in vivo* study of MI, we found that αCT11 (100 μM final concentration in the bloodstream) did not reduce the infarct size or EF% at D1 and D28 following intravenous administration (Figure 2A,B). Examination of αCT11 blood levels using mass spectrometry suggested that the peptide was completely degraded 10 min after injection, providing a likely explanation for the absence of cardioprotection (Figure 2C) relative to that observed in an *ex viv*o mouse model of cardiac I/R injury.^29^ Interestingly, when αCT11 was directly added into plasma isolated from whole blood and treated under different experimental conditions, it was found that (1) αCT11 degradation was proportional to the incubation time (Figure 3), (2) the breakdown rate of αCT11 was increased at higher (physiological) temperatures (Figure 4), and (3) degradation was decreased by the addition of PTase/PPTase inhibitors to the plasma (Figure 5). There were no significant differences in peptide degradation between males and females at either RT or 37 °C in the presence or absence of inhibitors (Figure S1). The underlying mechanisms associated with *in vivo* and *ex vivo* degradation of αCT11 are yet to be determined but are likely related to direct hydrolysis of the peptide by various proteases and phosphatases, such as aspartic and serine proteases (Figures 6 and S2 and Tables S1–S5) in the circulation. To the best of our knowledge, this is the first report examining the potential limiting factors that influence αCT11 degradation using both *in vivo* and *ex vivo* models. This study provides new insight into αCT11 degradation, and the findings may be used to prolong peptide half-life in the bloodstream, thus improving the efficiency of peptide-based therapy for patients with various diseases, such as cardiovascular disease. Moreover, while the issue of the instability of peptide therapeutics *in vivo* is generally known among those in the field^40^ and is well discussed in reviews of the literature,^25,41^ there are few concrete examples of the phenomenon in the primary literature as illustrated in the present study.

αCT1 was first reported in 2005 for its biological role in inhibiting the binding of zonula occludens 1 (ZO-1) to the C-terminus of Cx43 in cardiomyocytes.^42^ 4-year later, one of its variants, αCT11, was found to have the same or similar properties (e.g., membrane permeable) and biological functions (e.g., modulating Cx43 or ZO-1) as αCT1.^29^ In recent years, numerous studies, especially with αCT1, have been conducted to investigate αCT1′s roles in wound healing,^13,43^ cardiovascular protection,^29,41,44^ and as an adjunct therapy for cancer.^45−47^ In terms of αCT(1/11)’s therapeutic benefits for MI and HF, only a few studies using animal or isolated organs have been reported. In the cryo-injured ventricular model of mouse heart, O’Quinn et al. found that methylcellulose patches containing αCT1 adhered to injured heart tissue reduced colocalization of Cx43 and ZO-1, increased intercalated disk association of Cx43 at the injury border zone (IBZ), and showed reductions in cardiac arrhythmias and an increase in depolarization rates in treated hearts relative to controls.^48^ These beneficial effects were associated with αCT1-induced phosphorylation of Cx43 at Serine 368 on the CT of the protein.^48^ A recent study using isolated perfused mouse hearts subjected to I/R (20/40 min) injury revealed that infusion with either αCT1 or αCT11 promoted cardioprotection in the *ex vivo* model, as evidenced by significant increases in left ventricular contractile function.^29^

The above beneficial effects were not observed in the present study with I/R (40 min/open until D1 or D28) MI model of rats *in vivo*, where intravenous αCT11 (100 μM final concentration in the bloodstream) did not reduce the infarct size at either D1 or D28 following I/R injury (Figure 2). The main reasons for the different results between the two studies are likely related to the different experimental conditions and animal models used, such as the *ex vivo* model vs the *in vivo* model and αCT1 patches vs iv injection of αCT11. Under these conditions, the half-life of αCT1/αCT11 in blood that contains extremely complex components, including PTases,^25^ was likely to be significantly shorter than that of the well-defined simple Krebs-Henseleit buffer (no proteins/enzymes) used in the *ex vivo* study.^29^ Consistent with this hypothesis, in the present study, αCT11 was nearly undetectable in blood within 10 min of intravenous administration *in vivo*, potentially leaving the peptide without sufficient time to provide cardioprotective benefit.

The plasma of humans or other mammals (e.g., rats) is composed of about 92% water and 8% solids, such as enzymes (e.g., PTases, PPTases), nonenzymatic proteins (e.g., albumin), lipids (e.g., triglycerides), hormones (e.g., testosterone, estrogen), and ions/salts (e.g., Ca^2+^, NaCl).^49^ Among the 8% solid portion, albumin, globulin, fibrinogen, and transferrin account for 99% of the protein in plasma, with the remaining 1% consisting of PTases, PPTases, and coagulation factors.^50^ It is known that PTases play critical roles in various biological processes, such as protein/peptide degradation, signaling transduction, blood clotting, immune responses, and apoptosis,^51^ while PPTases are associated with, among other processes, signal transduction, dephosphorylation, cell cycling, metabolism, muscle contraction, and DNA repair.^52,53^ Due to their hydrolytic function, PTases in the bloodstream likely quickly degrade αCT11 (Figures 2C and 6) and shorten its half-life, thus reducing biological activity, which may be the main reason for the lack of cardioprotection observed in the present study. In contrast, in the previous studies using the *ex vivo* I/R model^29^ and the peptide patch approach,^48^ PTase activity was likely not a major factor. For example, in the Langendorff perfusion buffer or in the slowly dissolving methylcellulose patches, it is likely that sufficient local concentrations of αCT11 were maintained over time so that any effect was mediated.^29,48^ Indeed, in the latter study, αCT1 was detectable in cardiac tissues for up to 6 h following application of the patch to the cryo-injured heart.^48^ Interestingly, αCT11 was found to provide cardioprotection in an *in vivo* mouse model of I/R injury when 400 μg of peptide was injected intraperitoneally (i.p.).^54^ Further investigation is required to determine whether the differences in the administration route or other factors (e.g., rat and mouse differences) explain the inconsistent experimental outcomes observed in this earlier experiment vs the present study.

PTases/PPTases are enzymes and their biological activities can be affected by multiple factors, such as temperature, time, substance concentration, pH, and antagonists or agonists.^25,55^ In the present study, αCT11 degradation was highly time- and temperature-dependent in both male and female rats. The αCT11 concentration was reduced to 50% within 4–5 min *in vivo* and was almost undetectable at 10 min after tail vein injection (Figure 2). Similarly, but with delayed responses in *ex vivo* plasma, the αCT11 concentration decreased to 50% at about 10 min and was almost undetectable at 60 min in both males and females at 22 °C (Figure 3). Degradation rates appeared to be appropriate to the incubation temperature from 22 to 37 °C in both genders (Figure 4). These responses agree with commonly observed properties of enzyme activity.^56−59^ Based on the transition state theory, enzymes (here PTases) and their substrates (here αCT11) need to collide with each other while having a certain energy level to form an enzyme–substrate complex that will form products.^60^ As such, the PTases and PPTases involved in this study might reach their maximum “collision and energy transfer” within 30 min at 37 °C.

Because of their high affinity and high specificity, enzyme antagonists and agonists are probably the most forceful factors in the regulation of enzyme activities to decrease or increase the degradation of therapeutic peptides. As such, antagonists have the potential to increase the half-life of therapeutic peptides undergoing clinical testing.^61,62^ In lab research, PTase/PPTase inhibitor cocktails are commonly used together with cell lysis reagents to prevent degradation of extracted proteins for various protein analyses, such as Western blot.^18^ The present study found that the addition of a mixture of these inhibitory compounds significantly mitigated αCT11 degradation after 30 min incubation at both 22 and 37 °C and after 60 min incubation at 22 °C (Figure 5), leading to an overall increase of half-life to about 30–50 min at 37 °C, which could significantly accelerate therapeutic efficacy in protein or peptide-based therapies.^63^

However, the cocktail of inhibitors used in the present study is unlikely to be used for clinical management because of the likelihood of side effects from some, or all, of the components in such mixtures. For this reason, other strategies must be explored to reach the same goal of increasing the stability and half-life of therapeutic peptides.^25,63−65^ For example, fusing a peptide to the stable bacterial Rop (repressor of primer) protein or incorporation of two proline residues has been shown to significantly increase resistance to proteinase-induced degradation.^66^ Conjugating alpha1 proteinase inhibitor (α1PI) with poly(ethylene glycol) (PEG) at Cys (232) has also been observed to extend the *in vivo* half-life of α1PI in the bloodstream.^67^ Furthermore, *Enalapril* [ACE inhibitor, a small chemical drug to treat hypertension, kidney disease, and HF in the clinic] inhibits the degradation of BIO1211 (an anti-inflammatory peptide).^68^ Alternately, encapsulation of peptides in nanoparticles or extracellular vesicles may provide additional routes for stabilizing peptides, such as αCT11, in body fluids.^24,45^

While the biological activities of PTase/PPTases can be modulated by multiple factors,^25,55^ as demonstrated in the present study (e.g., time and temperature), no statistically significant differences in αCT11 degradation were found between males and females under our current experimental conditions (Figure S1), suggesting that sex may not play a role in regulating αCT11 degradation. Interestingly, sex hormones (e.g., estrogen, progesterone, testosterone) are known to widely regulate numerous biological processes, not only directing the development of a sexual system but also affecting the renin–angiotensin system^69^ and cardiac remodeling following MI.^70^ Whether sex hormones directly or indirectly affect the activities of proteases and phosphatases, thus modulating peptide degradation, remains to be investigated.

## Conclusions

In summary, the present study demonstrates that αCT11 was degraded faster in *in vivo* than in *ex vivo* models. The observed degradation rate was highly dependent on time and temperature but not gender. More importantly, PTase/PPTase inhibitors significantly delayed peptide degradation and prolonged the half-life of αCT11 at 20 and 37 °C in both genders. These findings indicate that PTases/PPTases in blood plasma play critical roles in promoting αCT11 degradation. Since the inhibitors only partially prevented peptide degradation, other limiting factors (e.g., physical clearance via kidney, enzymatic processes, metabolism in the liver) may also be involved. The complete set of mechanisms underlying the observed degradation remains unknown, and it is also not realistic or safe to use the chemical inhibitors employed in our *ex vivo* models in patients. As new drug delivery strategies (e.g., EVs and nanoparticles) emerge,^24,45^ αCT11 may be able to be loaded into EVs, for example, and be effectively protected from enzymatic degradation during intravenous injection or oral gavage delivery. Such approaches combining multiple methods may yield promising results in protecting small peptides *in vivo*, bringing them closer to clinical application and eventually being used as treatments for cardiovascular disorders, including MI and HF. Several critical limitations are also recognized from the present study: (1) Single (instead of mixtures) inhibitors specific for each type of PTase or PPTase should be individually tested in *in vivo*, *ex vivo*, and i*n vitro* models to identify the enzymes most responsible for αCT11 degradation, (2) the concentration of each inhibitor should be optimized in each model, and (3) the detailed pharmacokinetics of αCT11 and inhibitors also require further study. Despite these limitations, the present study provides new insight into the potential for rapid degradation of αCT11 in body fluids, such as blood and gastrointestinal fluids, while protective effects of PTase/PPTase inhibition on peptide stability may lead to the identification of new approaches to increase the half-life of therapeutics peptides based on our current finding with short peptides.

## Data Availability

All data are included in the article and in the supplemental files.

## Supporting Information Available

αCT11 stability in isolated rat plasma stored at 4 °C; ultraperformance liquid chromatography methods for αCT11 analysis; multiple reaction monitoring and mass spectrometry turning parameters for αCT11 quantification; mass spectrometry tuning parameters for αCT11 detection; proteases and phosphatases inhibitor cocktails; and αCT11 bonds targeted by proteases and phosphatases. (PDF)
