# Supplementary material for: Degradation of the α-Carboxyl Terminus 11 Peptide: In Vivo and Ex Vivo Impacts of Time, Temperature, Inhibitors, and Gender in Rat
Source: ACS Pharmacol Transl Sci. 2024 Apr 22;7(5):1624–36. doi: 10.1021/acsptsci.4c00120 (PMC11091968; doi:10.1021/acsptsci.4c00120)
Supplement: Supplementary file 1 — pt4c00120_si_001.pdf [file pt4c00120_si_001.pdf]

Supporting Information

Degradation of the α-Carboxyl Terminus 11 Peptide: *In Vivo* and *Ex Vivo* Impacts of Time, Temperature, Inhibitors, and Gender in Rat

Yagmur Tasdemiroglu[a#], McAlister Council-Troche[a#], Miao Chen[a], Benjamin Ledford[a], Russell A Norris[b], Steven Poelzing[c], Robert G Gourdie[c,*], and Jia-Qiang He[a,*]

[a]Department of Biomedical Sciences and Pathobiology, College of Veterinary Medicine, Virginia Tech, Blacksburg, VA 24061, USA.

[b]Department of Medicine, Medical University of South Carolina, Charleston, SC 29425, USA.

[c]Center for Vascular and Heart Research, Fralin Biomedical Research Institute, Virginia Tech, Roanoke, VA 24016, USA.

[#]These authors contributed equally to the work.

*Co-corresponding author: Dr. Gourdie is at the Center for Heart and Reparative Medicine Research, Fralin Biomedical Research Institute, Virginia Tech, 2 Riverside Circle, Roanoke, VA 24016, USA. Tel: 540-526-2095; Email: gourdier@vtc.vt.edu.

*Co-corresponding author: Dr. He is at the Department of Biomedical Sciences and Pathobiology, College of Veterinary Medicine, Virginia Tech, 225 Duck Pond Drive, Blacksburg, VA 24061, USA. Tel: 540-321-2032; Email address: jiahe@vt.edu.

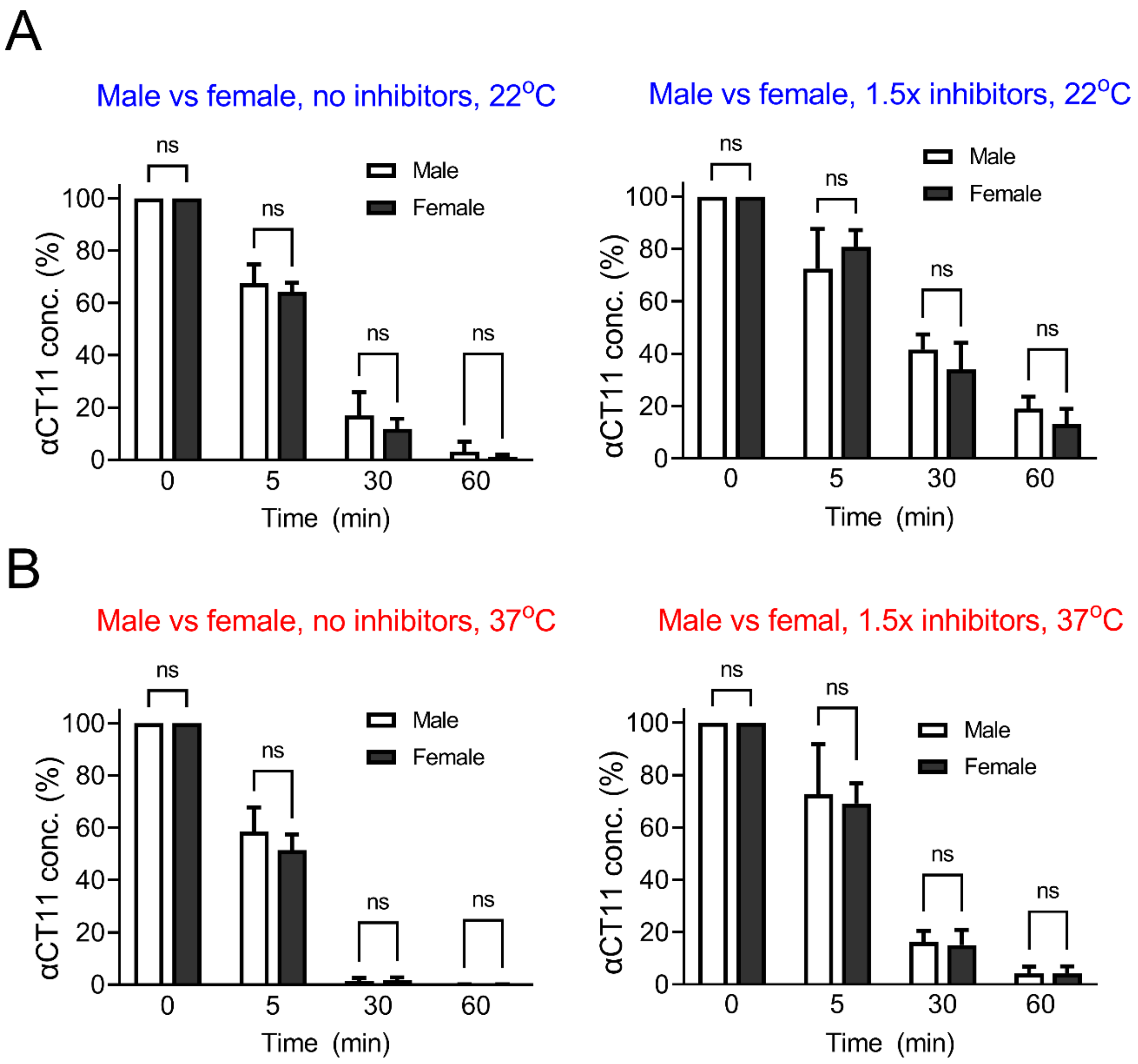

Figure S1. αCT11 degradation appears to be comparable between male and female. A) and **B)** Blood was collected from the left ventricular chambers of male (open bars) and female (black bars) rats under deep anesthesia. After being separated from the blood cells using a 4°C centrifuge, the resulting plasma was transferred into different tubes and αCT11 added to reach a final concentration of 100 μM in the presence or absence of 1.5x protease/phosphatase inhibitor cocktails. Samples were then maintained at 22°C (Panel **A**) or 37°C (Panel **B**) for 0, 5, 30, and 60 min, followed by measurement of plasma αCT11 using LC-MS/MS. The results are plotted as percent change (%) normalized to those at 0 min under each temperature. In both **Panel A** and **B**, a total of 9 replicates from 3 rats in each group were used under each temperature. Two-way ANOVA was used for statistical testing. ns: not significant. See Figures 1 to 5 for abbreviations defined previously.

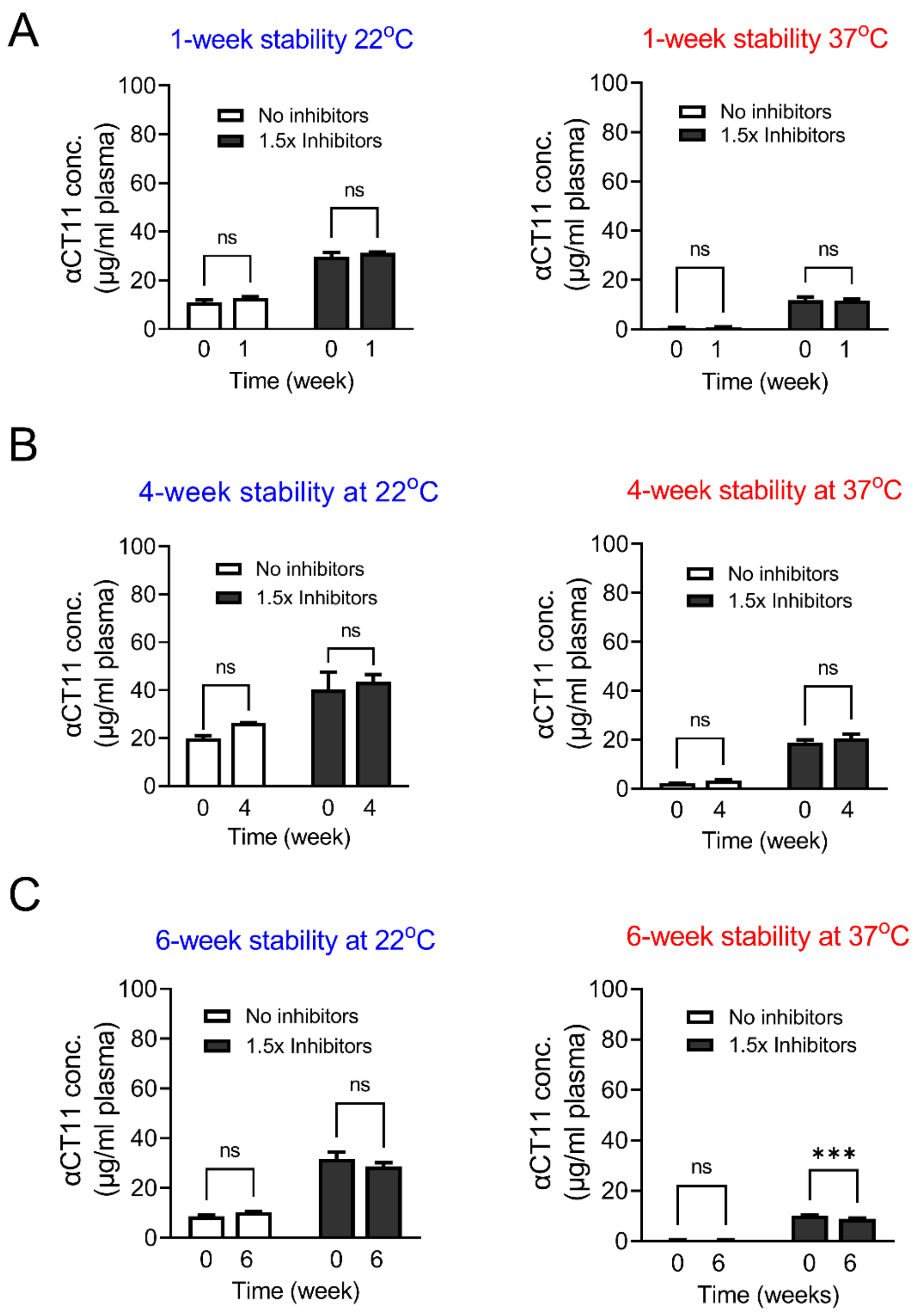

Figure S2. αCT11 stability in isolated rat plasma during various storage periods at 4°C. Blood was collected from the left ventricular chamber of male rats. After being separated from blood cells using a 4°C centrifuge, the resulting plasma was then transferred into different tubes and αCT11 was added to reach a final concentration of 100 μM in the presence or absence of 1.5x protease/phosphatase inhibitor cocktail. Following the initial measurements at Week 0 (*i.e.,* Day 0 when fresh plasma was isolated and αCT11 concentrations were measured at 0 min at 22°C and 37°C), the same samples used in each conduction were stored at 4°C for 1 week (**A**), 4 weeks (**B**), or 6 weeks (**C**) before being used again to measure the peptide concentrations. The results are plotted as percent change (%) of αCT11 normalized to those at Week 0 (Day 0 at 4°C). Two-way ANOVA was used for statistical testing. ns: no significant; *: p<0.05; ***: p<0.001. See Figures 1 to 6 and Figure 1S for the abbreviations defined previously.

Table S1. UPLC gradient method used for αCT11 analysis.

Time (mins)	%A solution (1%FA in H_2O)	%B solution (1%FA in ACN)
0.00	77	23
2.50	2	98
3.00	2	98
3.01	77	23
4.50	77	23

Abbreviations: α-carboxyl terminus 11: aCT11; ACN: acetonitrile; FA: formic acid; UPLC: ultra-performance liquid chromatography.

Table S2 . MRM transitions and specific mass spectrometry tuning parameters for the quantification of αCT11 peptide.

Analyte	Parent ion (amu)	Product ion (amu)	Cone energy (V)	Collision energy (eV)
αCT11 peptide	725.7 $[M+2H]^{2+}$	227.0	60	52
	725.7 $[M+2H]^{2+}$	237.2	24	48

Abbreviations: α-carboxyl terminus 11: aCT11; amu: atomic mass unit; eV: electron volts; $[M+2H]^{2+}$: mass (M) of doubly charged molecular ion; MRM: multiple reaction monitoring (a method used in mass spectrometry); V: voltage.

Table S3. Mass spectrometer tuning parameters for detecting αCT11 peptide.

Parameter	Value	Units
Capillary	1.50	kV
Cone	64	V
RF	2.50	V
Extractor	3.00	V
Source temperature	150	^oC
Desolvation temperature	500	^oC
Cone gas flow rate	50	liter/hour
Desolvation gas flow rate	750	liter/hour

Abbreviations: α-carboxyl terminus 11: aCT11; RF: radio frequency; kV: kilovolt; V: volt.

Table S4. Protease and phosphatase inhibitor cocktail and their major targets.

	Ingredient	MW (Da)	Conc. in cocktail (100x)	Targeted enzymes	Inhibitory mechanisms	IC_{50} or K_i	Refs
Protease Inhibitor Cocktail	AEBSF	239.5	0.11 mM	Serine proteases	Irreversible inhibition by reacting with the hydroxyl residue of the serine protease active site. The reaction forms a sulfonyl derivative that is stable for extended periods of time	Depends. When tested on K293 cells, IC_{50} = 1 mM. When tested on HS596 cells and SKN695 cells, IC_{50} = 300 μM.	1-3
	Aprotinin (Trasylol)	6511.4	0.08 mM	Serine proteases	It is a competitive inhibitor of trypsin, chymotrypsin, plasmin and kallikrein proteases. The reaction results in the formation of reversible enzyme-inhibitor complexes.	K_i = 0.2 to 1 nM against Trypsin 1 and 2; K_i = 0.17 μM against Chymotrypsin.	3-5
	Bestatin (Ubenimex)	308.4	5 mM	Aminopeptidases	Competitive, reversible inhibition of the enzyme active site.	Depends on the target enzyme and conditions. IC_{50} ranges from 0.01 to 100 μM.	3, 6
	E-64	357.4	1.5 mM	Cysteine proteases	The active site of E-64 irreversibly binds to the active thiol group in cysteine proteases, forming a thioether linkage.	K_i = 9 nM against papain.	3, 7, 8
	Leupeptin	475.6	2 mM	Serine and cysteine proteases	It acts as a transition state analogue and reversibly binds to its target proteases.	K_i = 3.5 nM against trypsin. K_i = 3.4 nM against plasmin.	3, 9, 10
	Pepstatin A	685.9	1.5 mM	Aspartic proteases	Reversible inhibition of aspartic proteases such as cathepsin D and pepsin.	IC_{50} = 21 nm against human cathepsin D; K_i = 0.01 nM.	3, 11
Phosphatase Inhibitor Cocktail	(-)-p-bromotetramisole oxalate	373.2	2.5 mM	Alkaline phosphatases	Non-specific inhibition of alkaline phosphatases.	N/A.	3, 12
	Cantharidin	196.2	500 μM	Serine/Threonine phosphatases	Dose-dependent inhibition. Competitively binds to the active sites of phosphatase targets.	IC_{50} = 0.47 μM against serine/threonine protein phosphatases; IC_{50} = 0.04 μM against serine/threonine protein phosphatases 2A.	3, 13, 14
	Imidazole (glyoxaline)	68.1	200 mM	Alkaline phosphatases	Uncompetitive inhibition. Imidazole interacts with the enzyme-substrate complex, forming an enzyme-inhibitor-substrate complex.	N/A.	3, 15
	Microcystin LR (MC-LR)	995.2	500 nM	PP1 and PP2A	Binds to the active sites of the phosphatase enzymes. For PP2A inhibition, MC-LR covalently binds to the Cys269 residue of the active site, inhibiting phosphatase activity.	IC_{50} = 0.3 to 2 nM against PP1 IC_{50} = 0.05 to 1 nM against PP2A.	3, 16, 17
	Sodium molybdate	205.9	115 mM	Acid and phosphoprotein phosphatases	Unclear.	N/A.	3, 18
	Sodium orthovanadate	183.9	100 mM	Alkaline, phosphatases, PTPs, and ATPases	Irreversible inhibition. Vanadate ion is a phosphate analogue which acts as a transitional state analogue and binds to protein tyrosine phosphatases.	IC_{50} = 100 μM against phosphatidylserine.	3, 19, 20
	Sodium tartrate	230.1 Da	400 mM	Acid phosphatases	Binds to the active site of acid phosphatases.	K_i = 1 μM against lysosomal and prostatic acid phosphatases.	3, 21
	Sodium Fluoride	42 Da	100 mM	Acid phosphatases	Irreversible inhibition. It is a phosphate analogue that competitively binds to acid phosphatases.	Ki = 10 to 100 μM against osteoblastic acid phosphatase.	3, 22

Abbreviations: AEBSF: 4-(2-aminoethyl) benzenesulfonyl fluoride hydrochloride; ATPase: adenosine 5′-triphosphatase; E-64: L-trans-3-carboxyoxiran-2-carbonyl-L-leucylagmatine; IC_{50}: half maximal inhibitory concentration; ; Ki: inhibitory constant; MW: molecular weight; N/A: not applicable; PP1 or 2A: protein phosphatase 1 or 2A; PTP: protein tyrosine phosphatase; K293: human embryonic kidney cell line; HS596: human HS683 glioma cell line; SKN695: human SK-N-SH neuroblastoma cell line.

(1) Citron, M.; Diehl, T. S.; Capell, A.; Haass, C.; Teplow, D. B.; Selkoe, D. J. Inhibition of amyloid beta-protein production in neural cells by the serine protease inhibitor AEBSF. *Neuron* **1996**, *17* (1), 171-179. DOI: 10.1016/s0896-6273(00)80290-1.

(2) Powers, J. C.; Asgian, J. L.; Ekici, O. D.; James, K. E. Irreversible inhibitors of serine, cysteine, and threonine proteases. *Chem Rev* **2002**, *102* (12), 4639-4750. DOI: 10.1021/cr010182v.

(3) Bimake.com. *Protease Inhibitor Cocktail (EDTA-Free, 100X in DMSO)* 2022. https://www.bimake.com/product/protease-inhibitor-cocktail.html (accessed).

(4) Belorgey, D.; Dirrig, S.; Amouric, M.; Figarella, C.; Bieth, J. G. Inhibition of human pancreatic proteinases by mucus proteinase inhibitor, eglin c and aprotinin. *Biochem J* **1996**, *313 (Pt 2)*, 555-560. DOI: 10.1042/bj3130555.

(5) Mahdy, A. M.; Webster, N. R. Perioperative systemic haemostatic agents. *Br J Anaesth* **2004**, *93* (6), 842-858. DOI: 10.1093/bja/aeh227.

(6) Scornik, O. A.; Botbol, V. Bestatin as an experimental tool in mammals. *Curr Drug Metab* **2001**, *2* (1), 67-85. DOI: 10.2174/1389200013338748.

(7) Matsumoto, K.; Mizoue, K.; Kitamura, K.; Tse, W. C.; Huber, C. P.; Ishida, T. Structural basis of inhibition of cysteine proteases by E-64 and its derivatives. *Biopolymers* **1999**, *51* (1), 99-107. DOI: 10.1002/(SICI)1097-0282(1999)51:1<99::AID-BIP11>3.0.CO;2-R.

(8) Arzanlou, M. Inhibition of streptococcal pyrogenic exotoxin B using allicin from garlic. *Microb Pathog* **2016**, *93*, 166-171. DOI: 10.1016/j.micpath.2016.02.010.

(9) Kurinov, I. V.; Harrison, R. W. Two crystal structures of the leupeptin-trypsin complex. *Protein Sci* **1996**, *5* (4), 752-758. DOI: 10.1002/pro.5560050420.

(10) Harris, C.; Jilek, J. L.; Sant, K. E.; Pohl, J.; Reed, M.; Hansen, J. M. Amino acid starvation induced by protease inhibition produces differential alterations in redox status and the thiol proteome in organogenesis-stage rat embryos and visceral yolk sacs. *J Nutr Biochem* **2015**, *26* (12), 1589-1598. DOI: 10.1016/j.jnutbio.2015.07.026.

(11) Leung, D.; Abbenante, G.; Fairlie, D. P. Protease inhibitors: current status and future prospects. *J Med Chem* **2000**, *43* (3), 305-341. DOI: 10.1021/jm990412m.

(12) Borgers, M.; Thone, F. The inhibition of alkaline phosphatase by L-p-bromotetramisole. *Histochemistry* **1975**, *44* (3), 277-280. DOI: 10.1007/BF00491496.

(13) Wang, G.; Dong, J.; Deng, L. Overview of Cantharidin and its Analogues. *Curr Med Chem* **2018**, *25* (17), 2034-2044. DOI: 10.2174/0929867324666170414165253.

(14) Ren, Y.; Kinghorn, A. D. Antitumor potential of the protein phosphatase inhibitor, cantharidin, and selected derivatives. *Bioorg Med Chem* **2021**, *32*, 116012. DOI: 10.1016/j.bmc.2021.116012.

(15) Brunel, C.; Cathala, G. Imidazole: an inhibitor of L-phenylalanine-insensitive alkaline phosphatases of tissues other than intestine and placenta. *Biochim Biophys Acta* **1972**, *268* (2), 415-421. DOI: 10.1016/0005-2744(72)90337-3.

(16) Kleppe, R.; Herfindal, L.; Doskeland, S. O. Cell Death Inducing Microbial Protein Phosphatase Inhibitors--Mechanisms of Action. *Mar Drugs* **2015**, *13* (10), 6505-6520. DOI: 10.3390/md13106505.

(17) Zhou, M.; Tu, W. W.; Xu, J. Mechanisms of microcystin-LR-induced cytoskeletal disruption in animal cells. *Toxicon* **2015**, *101*, 92-100. DOI: 10.1016/j.toxicon.2015.05.005.

(18) Pucell, A. G.; Hodges, J. C.; Sen, I.; Bumpus, F. M.; Husain, A. Biochemical properties of the ovarian granulosa cell type 2-angiotensin II receptor. *Endocrinology* **1991**, *128* (4), 1947-1959. DOI: 10.1210/endo-128-4-1947.

(19) Huyer, G.; Liu, S.; Kelly, J.; Moffat, J.; Payette, P.; Kennedy, B.; Tsaprailis, G.; Gresser, M. J.; Ramachandran, C. Mechanism of inhibition of protein-tyrosine phosphatases by vanadate and pervanadate. *J Biol Chem* **1997**, *272* (2), 843-851. DOI: 10.1074/jbc.272.2.843.

(20) Pelassy, C.; Breittmayer, J. P.; Aussel, C. Inhibition of phosphatidylserine synthesis during Jurkat T cell activation. The phosphatase inhibitor, sodium ortho-vanadate bypasses the CD3/T cell receptor-induced second messenger signaling pathway. *Eur J Biochem* **2000**, *267* (4), 984-992. DOI: 10.1046/j.1432-1327.2000.01081.x.

(21) Lindqvist, Y.; Schneider, G.; Vihko, P. Three-dimensional structure of rat acid phosphatase in complex with L(+)-tartrate. *J Biol Chem* **1993**, *268* (28), 20744-20746.

(22) Lau, K. H.; Farley, J. R.; Freeman, T. K.; Baylink, D. J. A proposed mechanism of the mitogenic action of fluoride on bone cells: inhibition of the activity of an osteoblastic acid phosphatase. *Metabolism* **1989**, *38* (9), 858-868. DOI: 10.1016/0026-0495(89)90232-1.

Table S5. The peptide bonds of αCT11 that are potentially targeted by proteases (PTases) and/or phosphatases (PPTases).

AA to AA bonds in αCT11	PTases		PPTases
	Enzyme subtype	Possible mechanisms underlying αCT11 degradation in the plasma	Potential mechanisms underlying αCT11 degradation in the plasma
C-terminus			
Isoleucine to Glutamic acid	aPTases<br>sPTases	It is believed that the aPTases polarize the carbonyl oxygen of the target peptide bond, and then form a tetrahedral intermediate. The aspartic acid in the active site acts as an electron donor, which result in a reaction that will break the target peptide bond[1]<br><br>In case of sPTases, the serine residue in their active site, along with histidine residue attacks the carbonyl group of the target peptide, promoting the formation of tetrahedral intermediate and acylenzyme intermediate. Finally, a water molecule is involved, causing the peptide bond to break and separates the enzyme from its substrate[2]	There is no distinct PPTase that directly contributes to PTases activation in the plasma. PPTases are relatively a novel area of research when compared with other enzymes like kinases. The reason why PPTase inhibitors are used in cell lysis is to prevent any dephosphorylation during the lysis process. There are PPTases that act on proteases however they are intracellular PPTases that act on intracellular PTases during apoptosis[3].<br><br>An example is phosphorylation of caspases (cysteine PTases that specifically act on aspartic acid bonds). A serine/threonine-protein kinase phosphorylates caspase-9, inhibiting apoptosis. Once caspase-9 gets dephosphorylated, apoptosis is promoted. A similar process is also seen with caspase-6, when being phosphorylated, it is inhibited, resulting in apoptosis inhibition; whereas it gets dephosphorylated, apoptosis is promoted. However, the phosphatase that promoted this dephosphorylation is unknown[4].
Glutamic acid to Leucine	aPTases<br>sPTases		
Leucine to Aspartic acid	aPTases<br>sPTases		
Aspartic acid to Aspartic acid	aPTases		
Aspartic acid to Proline	aPTases<br>sPTases<br>(*e.g.*, prolyloligopeptidase)		
Proline to Arginine	sPTases (e.g., prolyloligopeptidase)		
Arginine to Proline	sPTases<br>(*e.g.*, prolyloligopeptidase)		
Proline to Arginine	sPTases<br>(*e.g.*, prolyloligopeptidase)		
N-terminus			

Abbreviations: AA: amino acid; aPTases: aspartic proteases; PPTases: Phosphatases; PTases: Proteases; sPTases: serine proteases.

(1) Szecsi, P. B. The aspartic proteases. *Scand J Clin Lab Invest* **2011**, *52*, 5-22. Pereira, P. J.; Bergner, A.; Macedo-Ribeiro, S.; Huber, R.; Matschiner, G.; Fritz, H.; Sommerhoff, C. P.; Bode, W. Human beta-tryptase is a ring-like tetramer with active sites facing a central pore. *Nature* **1998**, *392* (6673), 306-311. DOI: 10.1038/32703.

(2) Hedstrom, L. Serine protease mechanism and specificity. *Chem Rev* **2002**, *102* (12), 4501-4524. DOI: 10.1021/cr000033x. Cunningham, D. F.; O'Connor, B. Proline specific peptidases. *Biochim Biophys Acta* **1997**, *1343* (2), 160-186. DOI: 10.1016/s0167-4838(97)00134-9.

(3) Alexander, D. R. The role of phosphatases in signal transduction. *New Biol* **1990**, *2* (12), 1049-1062. Kryczka, J.; Boncela, J. Proteases Revisited: Roles and Therapeutic Implications in Fibrosis. *Mediators Inflamm* **2017**, *2017*, 2570154. DOI: 10.1155/2017/2570154.

(4) Schwarz, S.; Hufnagel, B.; Dworak, M.; Klumpp, S.; Krieglstein, J. Protein phosphatase type 2Calpha and 2Cbeta are involved in fatty acid-induced apoptosis of neuronal and endothelial cells. *Apoptosis* **2006**, *11* (7), 1111-1119. DOI: 10.1007/s10495-006-6982-1. Velazquez-Delgado, E. M.; Hardy, J. A. Phosphorylation regulates assembly of the caspase-6 substrate-binding groove. *Structure* **2012**, *20* (4), 742-751. DOI: 10.1016/j.str.2012.02.003.
